# Reduction in Depressive Symptoms in People who Inject Drugs who Are Cured of Hepatitis C Virus Infection: The HERO Study

**DOI:** 10.1093/ofid/ofad498

**Published:** 2023-10-05

**Authors:** Irene Pericot-Valverde, Snehal S Lopes, Shadi Nahvi, James F Thrasher, Alison Karasz, Lynn E Taylor, Shruti H Mehta, Paula J Lum, Judith I Tsui, Kimberly Page, Judith Feinberg, Arthur Y Kim, Brianna L Norton, Julia H Arnsten, Sergio Fernandez-Artamendi, Moonseong Heo, Alain Litwin, HERO Research Group, Alain H Litwin, Moonseong Heo, Irene Pericot-Valverde, Hagan Walker, and Ashley Coleman, Shruti H Mehta, Courtney Borsuk, Brian Dickerson, Oluwaseun Falade-Nwulia, Michael Fingerhood, Taryn Haselhuhn, Angela Mason, Juhi Moon, Yngvild Olsen, and Vickie Walters, Arthur Y Kim, Jillian M Roche, William Schmitt, Virginia Lijewski, Anita Pitts, Syeda Raji, Taniya Silva, Fiona Evans, Hope Koene, Joelle Brown, Brianna Norton, Linda Agyemang, Julia Arnsten, Alison Karasz, Paul Meissner, Kiara Lora, Jennifer Hidalgo, Irene Soloway, Karen Jefferson, Joyce Wong, Andrea Kermack, Melissa Stein, Gilian Joseph, Karyn London, Lincoln Allen, Venecia Marte, Tatiana Vera, and Romy Alvarez, M Diane Mckee, Paula J Lum, Ellen S Stein, Anne F Luetkemeyer, Caycee Cullen, Gurjot Gill, Hannah Tierney, Scott Shapiro, Soraya Azari, Joanna Eveland, Daniel Berrner, Pauli Grey, and Jordan Akerley, Kimberly Page, Katherine Wagner, Herbert Davis, Cristina Murray-Krezan, Vanessa Jacobsohn, Jessica Anderson, Lynn E Taylor, Karen Tashima, Sophie Sprecht-Walsh, Aurielle Thomas, Melissa Hordes, Danielle McGregor, Patrick Duryea, and Kathryn Weenig, Judith I Tsui, Kendra L Blalock, Hyang Nina Kim, Meena S Ramchandani, Jocelyn R James, K Michelle Peavy, Paul Grekin, and Michael Ninburg, Judith Feinberg, Samuel Wilkinson, Danielle Thomas, Lacey Kelley, Andrea Calkins, Gabrielle Henry

**Affiliations:** Department of Psychology, Clemson University, Clemson, South Carolina, USA; Department of Public Health Sciences, Clemson University, Clemson, South Carolina, USA; Department of Medicine, Montefiore Medical Center/Albert Einstein College of Medicine, Bronx, New York, USA; Department of Health Promotion, Education, and Behavior, Arnold School of Public Health, University of South Carolina, Columbia, South Carolina, USA; Department of Family Medicine & Community Health, University of Massachusetts, Worcester, Massachusetts, USA; College of Pharmacy, University of Rhode Island . Kingston, Rhode Island, USA; Department of Epidemiology, Johns Hopkins Bloomberg School of Public Health, Baltimore, Maryland, USA; Department of Medicine, University of California, San Francisco, San Francisco, California, USA; Division of General Internal Medicine, University of Washington, Seattle, Washington, USA; Department of Internal Medicine, University of New Mexico Health Sciences Center, Albuquerque, New Mexico, USA; Department of Behavioral Medicine & Psychiatry and Department of Medicine, Infectious Diseases, West Virginia University School of Medicine, Morgantown, West Virginia, USA; Division of Infectious Diseases, Massachusetts General Hospital and Harvard Medical School, Boston, Massachusetts, USA; Department of Medicine, Montefiore Medical Center/Albert Einstein College of Medicine, Bronx, New York, USA; Department of Medicine, Montefiore Medical Center/Albert Einstein College of Medicine, Bronx, New York, USA; Department of Personality, Assessment, and Psychological Treatment, Universidad de Sevilla, Seville, Spain; Department of Public Health Sciences, Clemson University, Clemson, South Carolina, USA; Department of Psychology, Clemson University, Clemson, South Carolina, USA; Department of Medicine, University of South Carolina School of Medicine, Greenville, South Carolina, USA; Department of Medicine, Prisma Health, Greenville, South Carolina, USA

**Keywords:** DAA medication, HCV, PWID, depression

## Abstract

**Background:**

Depressive symptoms are prevalent among people who inject drugs (PWID) and people with hepatitis C virus (HCV). We examined changes in depressive symptoms among HCV-infected PWID following direct-acting antiviral treatments to evaluate whether these changes differed by history of depressive symptoms, substance use, or HCV treatment outcome.

**Methods:**

We conducted a secondary analysis of the HERO Study (NCT02824640), a pragmatic randomized clinical trial among PWID, to test the effectiveness of HCV care models. Depressive symptoms (primary outcome) were measured using the Patient Health Questionnaire (PHQ-9) at baseline, end of treatment (EOT), and at follow-up 12 and 24 weeks after EOT. Sustained virologic response (SVR) was defined as undetectable HCV RNA at ≥12 weeks following EOT. Baseline drug use was defined as having a positive urine screening test for amphetamine, methamphetamine, benzodiazepine, cocaine, cannabis, opiate, or oxycodone.

**Results:**

The sample (n = 498) was 72.3% male, 64.2% White, and on average 43.9 years old. In patients who achieved SVR (F(3432) = 4.58; *P* = .004) and those with drug use at baseline (F(3478) = 5.11; *P* < .01), PHQ-9 scores significantly declined over time, with scores lower at EOT and both follow-ups as compared with baseline. Mean PHQ-9 scores at EOT and follow-ups were significantly lower than at baseline, except for those with no depression or mild depression at baseline.

**Conclusions:**

This study showed that HCV treatment in PWID is associated with sustained declines in depression up to 24 weeks post-treatment among those who achieve SVR and that drug use does not interfere with improvement in depressive symptoms.

People who inject drugs (PWID) are particularly vulnerable to mental health disorders [1], with one of the most common disorders being depression. An estimated 42% of PWID experience moderate to severe depressive symptomatology, and ∼28.7% meet diagnostic criteria for major depressive disorder [2]. The prevalence of elevated depressive symptoms further increases among PWID when these present with other medical comorbidities, including hepatitis C virus (HCV) [3, 4]. Although elevated depressive symptoms do not necessarily interfere with HCV treatment intent, uptake, adherence, or sustained virologic response (SVR; equivalent to cure) [5], elevated symptoms have been associated with recent injection drug use (IDU) among HCV-infected PWID [4], which could negatively impact treatment success [6], thus evidencing the need for monitoring and assisting with depressive symptoms during HCV treatment.

Depressive symptoms have been shown to decrease during the course of HCV treatment with new, highly efficacious direct-acting antiviral (DAA) therapies [7–10]. However, prior research has not focused exclusively on active PWID who may suffer a greater burden of mental conditions such as depression [8–10], despite the fact that PWID are the population involved with the majority of ongoing HCV transmission worldwide [11]. Furthermore, these studies were unable to explore the impact of SVR status on post-SVR depressive symptoms as none included people who failed to achieve SVR.

To date, no study has examined the effect of HCV treatment with DAAs on depressive symptoms during the course of HCV treatment and post-treatment in a sample of PWID with recent drug use. This analysis aimed to address these gaps in the literature by exploring changes in depression severity among recently injecting HCV-infected PWID from baseline to the end of treatment (EOT) and post-treatment (12 and 24 weeks) among those with and without SVR, stratified by baseline symptom level and drug use.

## METHODS

### Parent Trial

The HERO study (NCT02824640) was a multisite (8 opioid treatment programs and 15 community health centers across 8 US cities) pragmatic randomized trial testing the effects of modified directly observed therapy (mDOT) or patient navigation (PN) care models with DAAs on HCV treatment outcomes among PWID with recent IDU. Eligibility criteria included (a) age 18–70 years; (b) current HCV infection; (c) aspartate transaminase, alanine transaminase, and platelets measured ≤12 months before entry; (d) self-reported active substance injection within 90 days of screening; (e) no previous DAA treatment; (f) willingness to receive sofosbuvir/velpatasvir; (g) willingness to be randomly assigned to either mDOT or PN; (h) if receiving methadone maintenance for opioid use disorder, willingness to attend the program ≥5 times per week; (i) able to provide written informed consent; and (j) English or Spanish fluency. Participants were ineligible if they were pregnant, breastfeeding, or diagnosed with hepatocellular carcinoma. Details about study procedures and findings have been published elsewhere [12]. In this study, we conducted a secondary analysis of a part of the per-protocol sample consisting of 498 participants who initiated treatment, completed a PHQ-9 questionnaire at baseline, complied with the assigned model of care, and had a determined SVR status.

### Patient Consent

Written informed consent was obtained from all participants. The study was conducted in accordance with the International Conference on Harmonization Good Clinical Practice (GCP) requirements and approved by the institutional review board of each institution (Clemson/Prisma Health, Johns Hopkins, Harvard Medical School, Albert Einstein College of Medicine, University of California San Francisco, University of New Mexico Health Sciences Center, University of Rhode Island, University of Washington, and West Virginia University).

### Measures

At baseline, participants completed a brief questionnaire collecting essential sociodemographic information. Depressive symptoms were measured using the Patient Health Questionnaire (PHQ-9) at baseline, EOT, and at follow-up 12 (SVR visit) and 24 weeks after EOT. Depression severity levels were determined based on PHQ-9 scores as follows: minimal to mild (0–9), moderate (10–14), moderately severe (15–19), and severe depression (≥20) [13]. Participants completed all self-reported questionnaires via Research Electronic Data Capture.

SVR was defined as having an HCV RNA level below the limit of quantitation (≤15 IU/mL) at ≥12 weeks following EOT. The time window for determination of SVR was 70–365 days after EOT. If unavailable by study blood draws, SVR was determined by clinical chart review. SVR was achieved by 92.2% (459/498) of participants.

Recent drug use at baseline was determined by self-report and a multidrug dip card that screened urine for amphetamine, methamphetamine, benzodiazepine, cocaine, cannabis, opiates, and oxycodone. IDU was also assessed via self-report.

Clinical characteristics including HIV coinfection, cirrhosis status, and HCV genotype were assessed by medical chart review. Information about current use of medications for opioid use disorder and previous HCV treatment was obtained by questionnaire.

### Statistical Methods

Participants’ baseline characteristics were compared across the baseline depression categories using analysis of variance/Kruskal-Wallis and chi-square/Fisher exact testing for continuous and categorical variables, respectively. Linear mixed-effects models were used to compare PHQ-9 scores across the study visits (baseline, EOT, and 12- and 24-week post-EOT follow-ups) by (i) depression severity level at baseline, including analyses stratified by SVR status; (ii) SVR status, including subgroup analyses among participants with a baseline PHQ-9 score ≥10; (iii) toxicology test results at baseline (any drug positive vs no drug positive). Following this, post hoc estimations of adjusted differences (adj. diff.; with 95% CIs) in mean PHQ scores from baseline to all subsequent visits were calculated by constructing pertinent linear contrasts from the fitted mixed-effects models. All analyses were adjusted for race, ethnicity, and both self-reported poly-drug use and drug test results for benzodiazepine at baseline; these covariates were significantly associated with the baseline depression severity level. All analyses were conducted using SAS 9.4. Test results with a 2-sided *P* value <.05 were declared statistically significant.

## RESULTS

### Participants’ Characteristics

The sample was 72.3% male, 64.2% White, 64.5% unemployed, and on average 43.9 years of age (Table 1). Fifty-two percent of the sample had minimal­­­ or mild levels of depression, 24.5% moderate, 13.7% moderately severe, and 9.8% severe as per PHQ-9 baseline scores. At baseline, the majority had a positive drug screen (96.4%), with the most commonly used substances being benzodiazepines (54.0%) and opiates (50.2%). The percentage of positive drug screens was 95.3%, 96.9%, and 95.7% at EOT, 12-week post-EOT follow-up, and 24-week post-EOT follow-up, respectively.

**Table 1.** Participants’ Baseline Characteristics

Characteristic	Overall n = 498 (100%)	Minimum–Mild Depression n = 259 (52.0%)	Moderate Depression n = 122 (24.5%)	Moderately Severe Depression n = 68 (13.7%)	Severe Depression n = 49 (9.8%)	*P*
Sociodemographics						
Age, mean (SD), y	43.9 (11.5)	44.3 (11.6)	44.7 (11.3)	41.7 (11.4)	42.8 (11.1)	.263
Gender						.304
Female	133 (26.7)	60 (23.2)	36 (29.5)	20 (29.4)	17 (34.7)	
Male	360 (72.3)	197 (76.1)	84 (68.9)	48 (70.6)	31 (63.3)	
Transgender	5 (1.0)	2 (0.8)	2 (1.6)	0 (0.0)	1 (2.0)	
Race						.047
White/Caucasian	308 (64.2)	148 (59.2)	70 (61.4)	53 (79.1)	37 (75.5)	
Black/African American	69 (14.4)	43 (17.2)	17 (14.9)	5 (7.5)	4 (8.2)	
Other	103 (21.5)	59 (23.6)	27 (23.7)	9 (13.4)	8 (16.3)	
Latino/Hispanic ethnicity						.035
No	386 (77.5)	197 (76.1)	88 (72.1)	61 (89.7)	40 (81.6)	
Yes	112 (22.5)	62 (23.9)	34 (27.9)	7 (10.3)	9 (18.4)	
Cohabitation status						.861
Single, separated, divorced, or widowed	437 (87.9)	228 (88.0)	107 (87.7)	60 (88.2)	42 (87.5)	
Married or living together	55 (11.1)	27 (10.4)	15 (12.3)	7 (10.3)	6 (12.5)	
Other	5 (1.0)	4 (1.5)	0 (0.0)	1 (1.5)	0 (0.0)	
Education						.358
Less than high school	116 (23.3)	64 (24.7)	29 (23.8)	13 (19.1)	10 (20.8)	
High school diploma or GED	188 (37.8)	87 (33.6)	54 (44.3)	30 (44.1)	17 (35.4)	
≥Some college	193 (38.8)	108 (41.7)	39 (32.0)	25 (36.8)	21 (43.8)	
Living stability						.948
Stable housing	253 (50.9)	135 (52.1)	61 (50.0)	33 (48.5)	24 (50.0)	
Unstable housing	244 (49.1)	124 (47.9)	61 (50.0)	35 (51.5)	24 (50.0)	
Employed						.003
Yes	176 (35.5)	103 (39.9)	40 (32.8)	27 (39.7)	6 (12.5)	
No	320 (64.5)	155 (60.1)	82 (67.2)	41 (60.3)	42 (87.5)	
Clinical-related characteristics						
PHQ-9 score, mean (SD)	10.0 (6.4)	4.9 (2.9)	12.0 (1.4)	16.8 (1.3)	22.1 (1.8)	<.001
Depression						.650
No	104 (42.3)	45 (44.1)	26 (36.1)	20 (45.5)	13 (46.4)	
Yes	142 (57.7)	57 (55.9)	46 (63.9)	24 (54.5)	15 (53.6)	
Anxiety						.817
No	139 (56.5)	61 (59.8)	39 (54.2)	23 (52.3)	16 (57.1)	
Yes	107 (43.5)	41 (40.2)	33 (45.8)	21 (47.7)	12 (42.9)	
Bipolar						.928
No	202 (82.4)	85 (83.3)	58 (81.7)	37 (84.1)	22 (78.6)	
Yes	43 (17.6)	17 (16.7)	13 (18.3)	7 (15.9)	6 (21.4)	
Schizophrenia						.545
No	229 (93.5)	97 (95.1)	67 (94.4)	40 (90.9)	25 (89.3)	
Yes	16 (6.5)	5 (4.9)	4 (5.6)	4 (9.1)	3 (10.7)	
PTSD						.049
No	206 (84.1)	93 (91.2)	55 (77.5)	37 (84.1)	21 (75.0)	
Yes	39 (15.9)	9 (8.8)	16 (22.5)	7 (15.9)	7 (25.0)	
Other						.829
No	150 (61.2)	60 (58.8)	43 (60.6)	28 (63.6)	19 (67.9)	
Yes	95 (38.8)	42 (41.2)	28 (39.4)	16 (36.4)	9 (32.1)	
SVR						.088
No	39 (7.8)	25 (9.7)	3 (2.5)	6 (8.8)	5 (10.2)	
Yes	459 (92.2)	234 (90.3)	119 (97.5)	62 (91.2)	44 (89.8)	
Treatment arm						.878
Modified directly observed therapy	247 (49.6)	130 (50.2)	57 (46.7)	34 (50.0)	26 (53.1)	
Patient navigation	251 (50.4)	129 (49.8)	65 (53.3)	34 (50.0)	23 (46.9)	
Clinical setting						.470
OTP	232 (46.6)	116 (44.8)	54 (44.3)	36 (52.9)	26 (53.1)	
CHC	266 (53.4)	143 (55.2)	68 (55.7)	32 (47.1)	23 (46.9)	
Opioid agonist therapy						.551
None	136 (27.3)	74 (28.6)	29 (23.8)	22 (32.4)	11 (22.4)	
Buprenorphine	60 (12.0)	31 (12.0)	19 (15.6)	6 (8.8)	4 (8.2)	
Methadone	302 (60.6)	154 (59.5)	74 (60.7)	40 (58.8)	34 (69.4)	
Previously received HCV treatment (non-DAA)						.713
No	473 (95.0)	248 (95.8)	114 (93.4)	65 (95.6)	46 (93.9)	
Yes	25 (5.0)	11 (4.2)	8 (6.6)	3 (4.4)	3 (6.1)	
Cirrhosis						.650
No	468 (94.0)	246 (95.0)	113 (92.6)	64 (94.1)	45 (91.8)	
Yes	30 (6.0)	13 (5.0)	9 (7.4)	4 (5.9)	4 (8.2)	
Genotype						.778
Type 1	272 (73.5)	144 (75.0)	65 (71.4)	39 (70.9)	24 (75.0)	
Type 2	32 (8.6)	15 (7.8)	10 (11.0)	5 (9.1)	2 (6.3)	
Type 3	60 (16.2)	31 (16.1)	15 (16.5)	8 (14.5)	6 (18.8)	
Type 4	3 (0.8)	1 (0.5)	0 (0.0)	2 (3.6)	0 (0.0)	
Mixed	3 (0.8)	1 (0.5)	1 (1.1)	1 (1.8)	0 (0.0)	
HIV coinfection (positive)						.422
No	289 (80.3)	154 (80.6)	62 (74.7)	46 (85.2)	27 (84.4)	
Yes	71 (19.7)	37 (19.4)	21 (25.3)	8 (14.8)	5 (15.6)	
Drug-related characteristics						
Last drug injection						.397
0–4 wk	370 (74.3)	182 (70.3)	95 (77.9)	53 (77.9)	40 (81.6)	
5–8 wk	85 (17.1)	49 (18.9)	18 (14.8)	12 (17.6)	6 (12.2)	
9–12 wk	43 (8.6)	28 (10.8)	9 (7.4)	3 (4.4)	3 (6.1)	
Times injecting drugs per day, mean (SD)	2.9 (2.7)	2.8 (2.5)	2.8 (2.1)	3.1 (2.5)	3.4 (4.5)	.759
Substances injected in the past 3 mo						
Mixture of cocaine and heroin	124 (26.2)	58 (23.6)	28 (24.8)	23 (34.3)	15 (31.3)	.270
Mixture of methamphetamine and heroin	108 (22.8)	55 (22.4)	31 (27.4)	10 (14.9)	12 (25.0)	.273
Heroin	382 (80.6)	194 (78.9)	95 (84.1)	49 (73.1)	44 (91.7)	.058
Methamphetamine	175 (36.9)	77 (31.3)	46 (40.7)	31 (46.3)	21 (43.8)	.057
Cocaine	139 (29.3)	61 (24.8)	34 (30.1)	24 (35.8)	20 (41.7)	.061
Crack	69 (14.6)	29 (11.8)	22 (19.5)	7 (10.4)	11 (22.9)	.065
Fentanyl	19 (43.2)	9 (34.6)	6 (54.5)	1 (25.0)	3 (100)	.130
Poly-substances	279 (58.9)	131 (53.3)	71 (62.8)	40 (59.7)	37 (77.1)	.015
Urine drug screen results positive at baseline visit						
Any drug	464 (96.7)	241 (95.6)	113 (96.6)	65 (100)	45 (97.8)	.396
Amphetamine	132 (27.5)	59 (23.4)	39 (33.3)	17 (26.2)	17 (37.0)	.101
Methamphetamine	149 (31.0)	66 (26.2)	43 (36.8)	21 (32.3)	19 (41.3)	.076
Benzodiazepine	259 (54.0)	123 (48.8)	66 (56.4)	38 (58.5)	32 (69.6)	.046
Cocaine	198 (41.3)	97 (38.5)	46 (39.3)	28 (43.1)	27 (58.7)	.077
THC/cannabis positive	239 (49.8)	114 (45.2)	64 (54.7)	35 (53.8)	26 (56.5)	.214
Opiate	241 (50.2)	120 (47.6)	59 (50.4)	35 (53.8)	27 (58.7)	.504
Oxycodone	128 (26.7)	66 (26.2)	27 (23.1)	17 (26.2)	18 (39.1)	.216

Data are presented as No. (%) unless otherwise indicated.

Abbreviations: CHC, ; DAA, direct-acting antiviral; HCV, hepatitis C virus; OTP, ; PHQ-9, Patient Health Questionnaire; PTSD, post-traumatic stress disorder; SVR, sustained virologic response.

### Changes in PHQ-9 Scores by SVR Status

**Figure 1.**
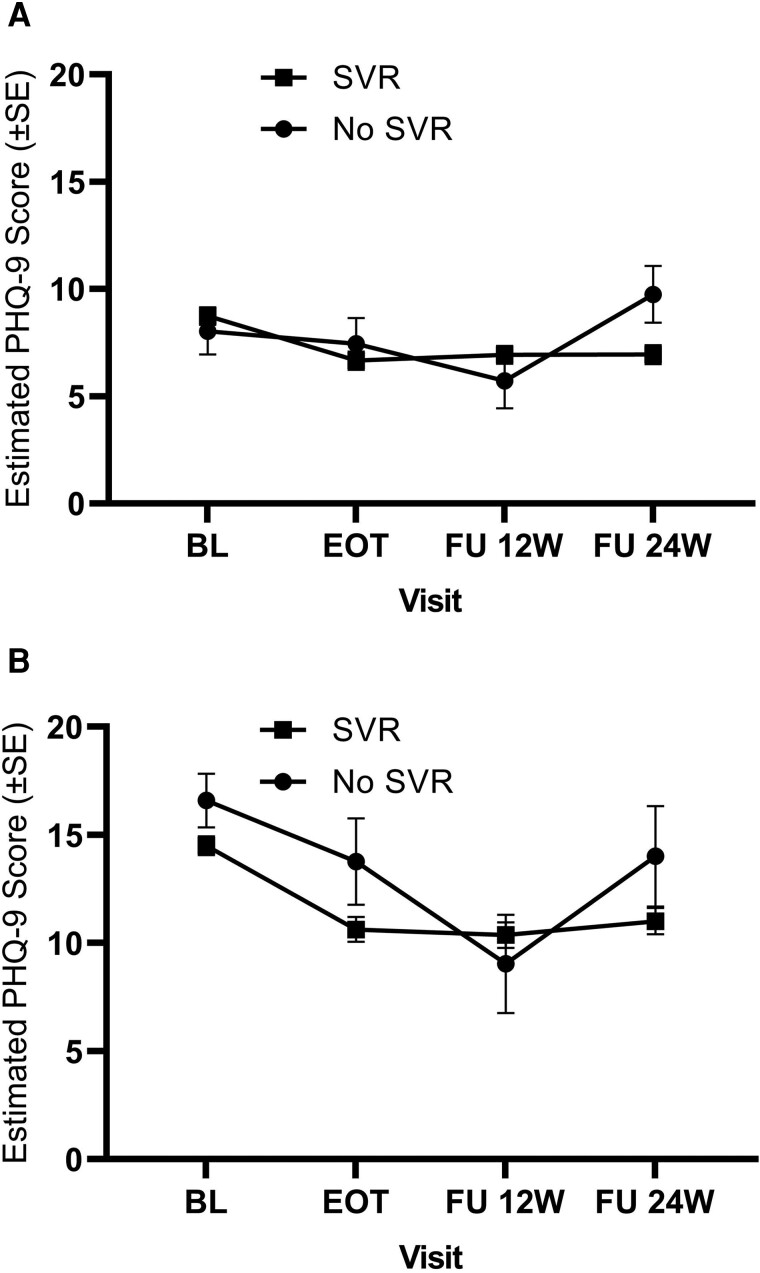
Estimates of PHQ-9 scores at each research visit by SVR status for overall participants (*A*, N = 498; SVR = 459 and no SVR = 39) and for participants with PHQ-9 scores ≥10 (*B*, N = 228; SVR = 223 and no SVR = 5). Error bars represent standard error. Abbreviations: BL, baseline; EOT, end of treatment; FU, follow-up; PHQ-9, Patient Health Questionnaire; SVR, sustained virologic response.

There was statistically significant effect modification by SVR status on PHQ-9 scores over time (F(3, 432) = 4.58; *P* = .004) (Figure 1*A*). In patients who achieved SVR (n = 459/N = 498), PHQ-9 scores significantly declined over time, with scores significantly lower at EOT and both post-EOT follow-ups (adj. diff. = −2.07, −1.80, −1.80; *ps* < .0001) as compared with baseline. In contrast, there were no statistically significant differences in PHQ-9 scores among participants who did not achieve SVR (n = 39/N = 498) at EOT and both post-EOT follow-ups (adj. diff. = −0.59, −2.32, 1.73) (Supplementary Table 1).

In the subgroup analyses among the participants with baseline moderate to severe (n = 239/N = 498) depressive symptoms (PHQ-9 score ≥10) (Figure 1*B*), there was a significant time effect (F(3, 201) = 9.81; *P* < .0001), but no significant effect of SVR status (F(1, 201) = 1.49; *P* = .224) or SVR status by time interaction (F(3, 201) = 1.47; *P* = .224). As compared with baseline, the depression scores were lower for the group with SVR (n = 255/N = 239) at each follow-up research visit (adj. diff = −3.88, −4.13, −3.50; *ps* ≤ .001) and for the group with no SVR (n = 14/N = 239) at 12-week post-EOT follow-up only (adj. diff. = −7.55; *P* < .001) (Supplementary Table 1).

### Changes in PHQ-9 Scores by Baseline Depression Severity Levels and Stratified by SVR Status

**Figure 2.**
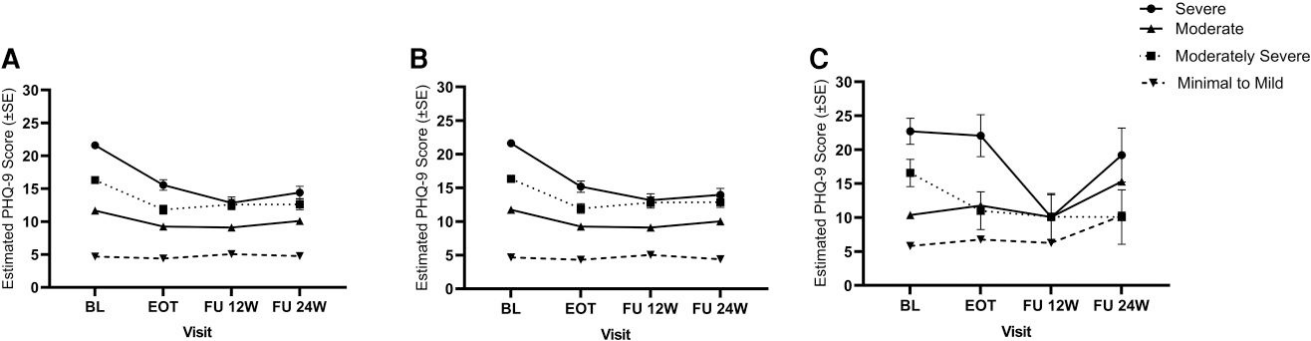
Estimates of PHQ-9 scores at each research visit for the overall sample (*A*, N = 498; severe = 49, moderately severe = 68, moderate = 122, minimal to mild = 259), participants who achieved SVR (*B*, N = 459; severe = 44, moderately severe = 62, moderate = 119, minimal to mild = 234), and participants who did not achieve SVR (*C*, N = 39; severe = 5, moderately severe = 6, moderate = 3, minimal to mild = 25). Error bars represent standard error. Abbreviations: BL, baseline; EOT, end of treatment; FU, follow-up; PHQ-9, Patient Health Questionnaire; SVR, sustained virologic response.

There was a significant baseline depression group by time interaction effect (F(9, 430) = 15.19; *P* < .0001), indicating that the changes in PHQ-9 scores differed by baseline depression severity levels (Figure 2*A*). PHQ-9 scores at EOT and both post-EOT follow-ups were significantly lower than at baseline within participants with moderate (n = 122/N = 498; adj. diff. = −2.43, −2.59, *−*1.58; *ps* < .001), moderately severe (n = 68/N = 498; adj. diff. = −4.50, −3.72, *−*3.71; *ps* < .001), and severe levels (n = 49/N = 498; adj. diff. = −6.02, −8.75, *−*7.18; *ps* < .001) of baseline depression. No significant changes in PHQ-9 scores were observed in participants with minimal to mild levels of baseline depression (n = 259/N = 498) between baseline and any of the follow-up research visits (adj. diff. = −0.28, 0.39, 0.10) (Supplementary Table 1).

In the subgroup analyses among participants with SVR (n = 459/N = 498) (Figure 2*B*), there was a significant baseline depression group by time interaction effect (F(9, 391) = 13.37; *P* < .0001), demonstrating that changes in PHQ-9 scores over research visits were different between the baseline depression levels. The mean PHQ-9 scores at EOT and follow-ups were significantly lower than at baseline for participants with moderate (n = 119/N = 459; adj. diff. = −2.50, −2.64; *ps* < .001, *−*1.73; *P* < .01), moderate–severe (n = 62/N = 459; adj. diff. = −4.41, −3.52, *−*3.46; *ps* < .001), and severe (n = 44/N = 459; adj. diff. = −6.45, −8.46, *−*7.66; *ps* < .001) levels of baseline depression. There was no statistically significant change in mean PHQ-9 scores among those with mild–moderate baseline depression levels (n = 234/N = 459; adj. diff. = −0.35, 0.38, −0.27) (Supplementary Table 1).

Also, in the subgroup analyses among those who did not achieve SVR (n = 39/N = 498), there was a significant baseline depression group by time interaction effect (F(9, 30) = 3.64; *P* = .004) (Figure 2*C*). Post hoc analyses showed that participants with the baseline minimal–mild depression level (n = 25/N = 39) increased their PHQ-9 scores from baseline to 24-week follow-up (adj. diff. = 4.48; *P* < .05). Those with the baseline moderate depression level (n = 3/N = 39) did not show a change in PHQ-9 scores from baseline to any follow-up research visit. PHQ-9 scores decreased from baseline to EOT and 12-week post-EOT follow-up (adj. diff. = −5.56 and −6.43; *P* < .05) for participants with moderately severe (n = 6/N = 39) baseline depression and decreased from baseline to 12-week post-EOT follow-up for participants with severe (n = 5/N = 39) baseline depression (adj. diff. = −12.66; *P* < .001) (Supplementary Table 1).

### Changes in PHQ-9 Scores by Drug Use at Baseline

There was a significant time effect (F(3, 432) = 4.35; *P* = .005) but no baseline drug use by time effect (F(3, 432) = 2.00; *P* = .114). In participants with a positive toxicology test at baseline (n = 464/N = 480), PHQ-9 scores at EOT and post-EOT follow-ups were significantly lower than at baseline (adj. diff. = −1.93, −1.91, −1.61; *ps* < .001). In participants with a negative toxicology test at baseline (n = 16/N = 480), PHQ-9 scores were lower at EOT compared with baseline (adj. diff. = −3.13; *P* < .05) but not at 12- and 24-week post-EOT follow-ups (adj. diff. = 0.97, −0.40) (Supplementary Table 1).

## DISCUSSION

In this sample of PWID, depressive symptoms substantially decreased at the end of HCV DAA treatment, with these reductions remaining up to 24 weeks post-treatment, but only among those who achieved SVR. When stratified by depression severity at baseline, all participant groups with moderate to severe baseline depression symptoms who achieved SVR experienced sustained reductions in depressive symptoms after treatment. Our results are consistent with those of other studies [7–10], in which PWID receiving DAA treatment experienced reductions in depressive symptoms. This study is among the first to show that changes in depressive symptoms may vary among PWID from pretreatment to end of treatment and for up to 24 weeks, whether or not SVR was achieved.

Depression among people with HCV is postulated to be an extrahepatic condition derived from the viral infection, which may be caused via different pathways, including cerebral or systemic inflammation or alterations in neurotransmitter circuits [14]. Symptoms that frequently co-occur with HCV infection (eg, sleep disturbances, fatigue) also might contribute to depression. Regardless of potential mechanisms underlying depression among people with HCV, our results suggest that depression persists in those PWID with HCV. These results further demonstrate that providing HCV treatment with DAAs not only cures HCV among PWID but also reduces depressive symptoms. Further research to be conducted could examine whether it is the cure of HCV and removal of the virus from the brain, cure of HCV leading to relief of the psychological burden of living with HCV, or a combination of these factors that facilitates the reductions in depression [15].

The finding that depressive symptoms significantly decreased after treatment completion and remained low for up to 24 weeks among those who were cured of HCV aligns with that of an earlier study showing that people with HCV who successfully achieved SVR experienced reductions in depressive symptoms during and up to 24 weeks post-treatment [7]. However, our study is the first to evaluate this issue among only active PWIDs. Almost all of our participants were actively using drugs at baseline, with 96.4% of participants testing positive for any drug toxicology (vs 49.6% in the prior study). Therefore, our study results suggest that recent and ongoing drug use does not hinder reductions in depressive symptoms during the course of HCV treatment, providing further support for treating HCV even among PWID who are actively injecting. Indeed, facilitating access to DAA HCV treatment is not only important to cure HCV in PWID, but also to improve their mental health.

It is also notable that patients who did not achieve SVR experienced an increase in depressive symptoms between the 12- and 24-week post-treatment follow-up. This is a novel finding that was not reported in earlier published studies [7–10] because these did not include a sample of people who failed to achieve SVR, and therefore they were unable to assess the impact of not achieving HCV cure on depressive symptoms. While the cause for this finding is unknown, there are potential explanations. Patients were not aware of their HCV status at the 12-week post-treatment follow-up (SVR visit), but they were at the 24-week post-treatment follow-up. It is possible that knowledge of their HCV status may have had an impact on their depressive symptoms. In this regard, previous studies have shown that HCV status awareness may influence self-reported health outcomes among PWID [16, 17] and that there are benefits beyond cure. Psychosocial support seems to be an important area for intervention among patients after receiving their HCV status after DAA treatment, especially among those who do not achieve HCV cure.

There are several limitations to this study that should be considered when interpreting the study findings. First, the HERO cohort was predominately PWID living in urban settings, limiting the generalizability of these findings to PWID living in more rural areas. Second, given that depressive symptoms were not assessed during treatment in the HERO study, we were not able to explore at which point during the treatment period depressive symptoms might have decreased. Third, this study is a secondary data analysis, and other factors that may influence depressive symptoms or their course, such as sex [18], were not included in the parent trial. Finally, the depression profiles were obtained using data from the PHQ-9 rather than using the DSM-5-TR criteria. Future studies might explore depression symptom profiles among HCV-infected PWID using the recently released DSM-5-TR criteria.

## CONCLUSIONS

In summary, this secondary data analysis of a multisite pragmatic trial involving a large sample of actively injecting PWID who were treated with DAAs found that DAA treatment leading to cure is associated with decreases in depressive symptoms among those with the most severe depression profiles for up to 24 weeks post-treatment. Additionally, we found that recent drug use does not interfere with the reductions of depressive symptoms seen following completion of HCV treatment. HCV cure among PWID presents benefits beyond cure in terms of improvements in depressive symptoms, which may improve quality of life and well-being. HCV treatment should never be withheld because of co-occurring depression or substance use.

## Supplementary Material

ofad498_Supplementary_Data

## Supplementary Data

Supplementary materials are available at *Open Forum Infectious Diseases* online. Consisting of data provided by the authors to benefit the reader, the posted materials are not copyedited and are the sole responsibility of the authors, so questions or comments should be addressed to the corresponding author.
