# Supplementary material for: Reduction in Depressive Symptoms in People who Inject Drugs who Are Cured of Hepatitis C Virus Infection: The HERO Study
Source: Open Forum Infect Dis. 2023 Oct 5;10(11):ofad498. doi: 10.1093/ofid/ofad498 (PMC10644781; doi:10.1093/ofid/ofad498)
Supplement: ofad498_Supplementary_Data [file ofad498_supplementary_data.docx]

Supplemental Table 1. Adjusted differences in mean PHQ-9 scores from baseline to EOT, FU12 weeks, and FU 24 weeks stratified by SVR status, baseline severity levels, and baseline urine toxicology results.

|  |  | Mean difference in PHQ-9 scores from baseline (95% CI) | | | | | |
| --- | --- | --- | --- | --- | --- | --- | --- |
| Sample | Stratifying Variables | EOT | | FU 12 Weeks | | FU 24 Weeks | |
|  | SVR Status |  |  |  |  |  |  |
| Total Sample | *SVR* | -2.07 (-2.60, -1.54) | *** | -1.80 (-2.45, -1.16) | *** | -1.80 (-2.44, -1.17) | *** |
|  | *No SVR* | -0.59 (-2.57, 1.40) |  | -2.32 (-4.70, 0.06) |  | 1.73 (-0.61, 4.06) |  |
| Among PHQ-9 ≥ 10 | *SVR* | -3.88 (-4.72, -3.03) | *** | -4.13 (-5.12, -3.14) | *** | -3.50 (-4.51, -2.48) | *** |
|  | *No SVR* | -2.83 (-6.40, 0.75) |  | -7.55 (-11.95, -3.15) | *** | -2.58 (-7.08, 1.93) |  |
|  | Baseline Depression Severity: | |  |  |  |  |  |
| Total Sample | *Minimal to Mild* | -0.28 (-0.94, 0.38) |  | 0.39 (-0.38, 1.17) |  | 0.10 (-0.70, 0.90) |  |
|  | *Moderate* | -2.43 (-3.42, -1.45) | *** | -2.59 (-3.75, -1.43) | *** | -1.58 (-2.79, -0.37) | * |
|  | *Moderately severe* | -4.50 (-5.75, -3.25) | *** | -3.72 (-5.24, -2.20) | *** | -3.71 (-5.23, -2.19) | *** |
|  | *Severe* | -6.02 (-7.53, -4.52) | *** | -8.75 (-10.51, -7.00) | *** | -7.18 (-8.98, -5.38) | *** |
| SVR | *Minimal to Mild* | -0.35 (-1.04, 0.33) |  | 0.38 (-0.43, 1.19) |  | -0.27 (-1.08, 0.54) |  |
|  | *Moderate* | -2.50 (-3.50, -1.50) | *** | -2.64 (-3.82, -1.47) | *** | -1.73 (-2.91, -0.55) | ** |
|  | *Moderately severe* | -4.41 (-5.73, -3.09) | *** | -3.52 (-5.09, -1.94) | *** | -3.46 (-4.99, -1.94) | *** |
|  | *Severe* | -6.45 (-8.02, -4.87) | *** | -8.46 (-10.29, -6.63) | *** | -7.66 (-9.49, -5.82) | *** |
| No SVR | *Minimal to Mild* | 0.95 (-1.42, 3.31) |  | 0.45 (-2.33, 3.22) |  | 4.48 (1.01, 7.94) | * |
|  | *Moderate* | 1.41 (-5.16, 7.97) |  | -0.30 (-8.20, 7.59) |  | 4.95 (-7.58, 17.48) |  |
|  | *Moderately severe* | -5.56 (-9.72, -1.40) | * | -6.43 (-12.58, -0.28) | * | -6.49 (-13.92, 0.93) |  |
|  | *Severe* | -0.66 (-5.88, 4.55) |  | -12.66 (-18.91, -6.40) | *** | -3.51 (-11.00, 3.97) |  |
|  | Baseline drug use: |  |  |  |  |  |  |
| Total Sample | *No drug positive* | -3.13 (-6.06, -0.21) | * | 0.97 (-2.51, 4.44) |  | -0.40 (-4.16, 3.37) |  |
|  | *Any drug positive* | -1.93 (-2.45, -1.41) | *** | -1.91 (-2.54, -1.28) | *** | -1.61 (-2.24, -0.98) | *** |
| Note: EOT: End of treatment; FU: Follow-up; PHQ-9: Patient Health Questionnaire-9; SVR: Sustained virologic response; Statistical significance levels: <.05*, <.01**, <.001***. Mixed effects models were used. Covariates in all models included race, ethnicity, self-reported poly-drug use at baseline, and baseline urine drug test for benzodiazepine. | | | | | | | |
